# First experiences of immersive virtual reality as an additional learning tool in curricular teaching for orthopedic and trauma surgery

**DOI:** 10.1007/s00132-025-04697-6

**Published:** 2025-08-12

**Authors:** Tobias Schöbel, Leonard Schuschke, Yasmin Youssef, Christoph-E. Heyde, Daisy Rotzoll, Georg Osterhoff, Jan Theopold

**Affiliations:** 1 Department of Orthopedics, Trauma and Plastic Surgery, University of Leipzig, Liebigstraße 20, 04103 Leipzig, Germany; 2 Skills and Simulation Centre LernKlinik Leipzig, Faculty of Medicine, University of Leipzig, Liebigstraße 23/25, 04103 Leipzig, Germany

**Keywords:** Augmented reality, Simulation training, Orthopedic surgery, Surgical education, Medical education, Augmented Reality, Simulationstraining, Orthopädische Chirurgie, Chirurgische Ausbildung, Medizinische Ausbildung

## Abstract

**Introduction:**

Immersive virtual reality (iVR) simulators have been introduced for skills training in various medical disciplines. While iVR simulators have been shown to improve technical skills, they are currently not well established in curricular teaching for medical students. The aim of this study was to demonstrate the implementation of an iVR operating theater in addition to conventional bedside teaching in orthopedic and trauma surgery and to evaluate user feedback regarding expectations, acceptance and limitations.

**Methods:**

Medical students must complete a week of bedside teaching in orthopedic and trauma surgery in the fourth year. Of these students one third (*n* = 56) were given the opportunity to attend an iVR operation theater using Oculus Quest 2 headsets and controllers and the PrecisionOS software. Participants were able to train several surgical procedures. User feedback was obtained before and after the obligatory course by two questionnaires and compared statistically using the Mann-Whitney-U-test or Fishers’ exact t‑test.

**Results:**

All students had high expectations regarding the implementation of iVR training in the course. The participants spent a mean time of 3.3 h per week using the iVR tool in addition to the regular course content. All students stated that iVR training was a useful addition to bedside teaching in orthopedic and trauma surgery. Most participants (87.0%) thought that VR applications will play a role in their future career. Motion sickness occurred in half of the participants. The main symptoms that were described were dizziness (37.0%), nausea (33.3%) and headaches (25.9%). Students who received an iVR tool rated the bedside course better than students who did not receive the iVR tool.

**Conclusion:**

The iVR training can be easily implemented into the curriculum of medical students. The high expectations of the participants were fulfilled and the students wished iVR to be a regular part of the obligatory courses in orthopedic and trauma surgery.

**Graphic abstract:**

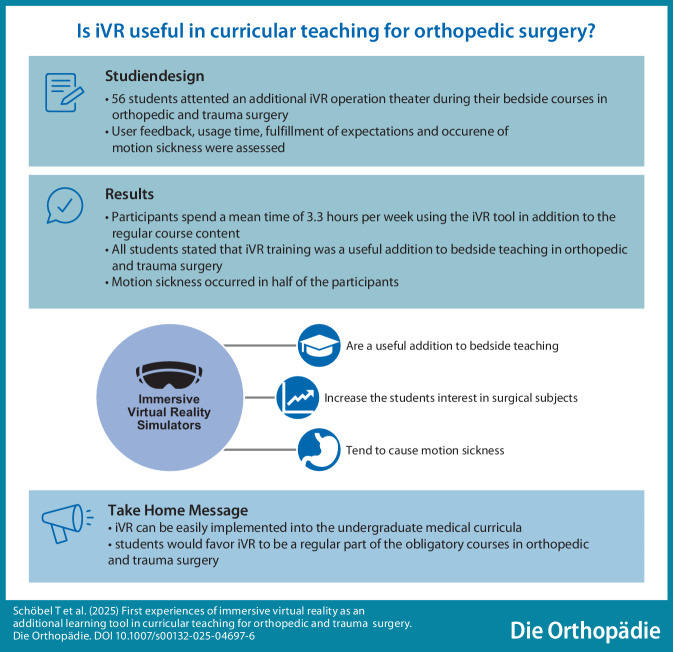

## Abstract (de)

**Einleitung:**

Immersive-Virtual-Reality-Simulatoren (iVR) wurden für das Training von Fähigkeiten in verschiedenen medizinischen Disziplinen eingeführt. Während iVR-Simulatoren nachweislich die technischen Fertigkeiten verbessern, sind sie derzeit in der Lehre für Medizinstudierende nicht gut etabliert. Ziel dieser Studie war es, die Implementierung eines iVR-Operationssaals als Ergänzung zum konventionellen Unterricht am Krankenbett in der Orthopädie und Unfallchirurgie zu demonstrieren und das Feedback der Nutzer hinsichtlich Erwartungen, Akzeptanz und Einschränkungen zu bewerten.

**Methoden:**

Medizinstudierende müssen in ihrem vierten Studienjahr eine Woche Unterricht am Krankenbett in Orthopädie und Unfallchirurgie absolvieren. Ein Drittel (*n* = 56) dieser Studierenden erhielt die Möglichkeit, an einem iVR-Operationssaal teilzunehmen, wobei Oculus Quest 2‑Headsets und -Controller sowie die PrecisionOS-Software verwendet wurden. Die Teilnehmenden konnten mehrere chirurgische Eingriffe trainieren. Das Feedback der Benutzer wurde vor und nach dem obligatorischen Kurs mit zwei Fragebögen eingeholt und statistisch verglichen.

**Ergebnisse:**

Alle Studierenden hatten hohe Erwartungen an die Implementierung der iVR-Technik in ihrem Kurs. Die Teilnehmenden verbrachten durchschnittlich 3,3 h pro Woche mit dem iVR-Tool zusätzlich zu den regulären Kursinhalten. Alle Studierenden gaben an, dass die iVR-Schulung eine nützliche Ergänzung zum Unterricht am Krankenbett in der Orthopädie und Unfallchirurgie darstellte. Die meisten Teilnehmenden (87,0 %) gaben an zu glauben, dass VR-Anwendungen eine Rolle in ihrer zukünftigen Karriere spielen werden. Bei der Hälfte der Teilnehmenden trat Motion Sickness auf. Als Hauptsymptome wurden Schwindel (37,0 %), Übelkeit (33,3 %) und Kopfschmerzen (25,9 %) beschrieben. Studierende, die den Kurs mit dem iVR-Tool bestritten, bewerteten den Kurs am Krankenbett besser als Studierende, die kein iVR-Tool erhielten.

**Schlussfolgerung:**

iVR kann leicht in den Lehrplan für Medizinstudenten integriert werden. Die hohen Erwartungen der Teilnehmenden wurden erfüllt, und die Studierenden wünschten sich, dass iVR ein regelmäßiger Bestandteil der obligatorischen Kurse in Orthopädie und Unfallchirurgie wird.

## Introduction

Surgical simulations are used for skills training in various medical disciplines and have become an integral component of medical education in recent years [9, 21]. Because the use of human cadavers as a long-established gold standard in surgical training is associated with many limitations, virtual reality (VR) simulation has been explored as an alternative for surgical training enabling the training of procedural and clinical skills in a psychologically safe environment [9, 17]. Consequently, VR technologies are currently being used in orthopedic and trauma training simulators to increase surgical accuracy, to improve outcomes and to reduce the risk for complications [34–37]. Furthermore, the multifactorial benefits of using VR simulation in orthopedic and trauma training include objective skills assessment, no compelling need for supervision, minimal disposable costs, high accessibility and the ability to translate learned skills to patient care [9]. Additionally, multiple studies suggest that the use of VR during orthopedic residency programs improves haptic surgical skills and is effective in internalizing surgical procedures [7, 8, 18, 22, 25, 29].

Improvements in technology allowed the development of immersive virtual reality (iVR), which can be defined as a fully virtual interactive simulation with a 3-dimensional (3D) environment projected onto a head-mounted display (HMD), enabling a 360° visual immersion and real-time manipulation of virtual items. It offers all advantages of conventional VR, while operating on low-cost, mobile and commercially available hardware [21, 26, 32].

Although there is good evidence that training on VR simulators improves technical skills, its use in orthopedic and trauma training programs is lower when compared to other surgical specialties [1, 10]. Training simulators are only available in a few residency programs [36] and only one study demonstrated the use of VR in curricular education in orthopedic and trauma surgery for medical students [13]. As cadaver training was negatively impacted by the COVID-19 pandemic [30] and the advantages of iVR have been described extensively in the current literature [7, 8, 18, 21, 22, 25, 29], not using iVR in student teaching seems peculiar. Therefore, the goal of this study was to demonstrate how iVR can be implemented into curricular teaching of medical students in the field of orthopedic and trauma surger, and to evaluate the expectations, acceptance and perceived limitations of the participants.

## Methods

### Technical equipment

In this study six Oculus Quest 2 headsets and corresponding controllers (Reality Labs, Meta Platforms, Menlo Park, USA) were used as a stand-alone head-mounted display. To ensure anonymization, preconfigured meta-accounts were provided for all six HMDs. PrecisionOS Platform Version 3.0 (PrecisionOS Technologies, Vancouver, Canada) was used as software. With the HMDs and the software, the participants were able to train a variety of surgical procedures in a realistic surgical scenario (3D operating room) on their own. Surgical complications (bleeding, nerve injuries, soft tissue damage, etc.) were not included in the applications. The available courses included, among others, the following surgical procedures:

1. proximal femoral nail fixation,
2. anterior hip approach for total hip arthroplasty,
3. total knee arthroplasty,
4. reverse shoulder arthroplasty system,
5. Infinity knee system anteromedial for ACL reconstruction.

### Curricular implementation

In the medical curriculum at Leipzig University, all medical students must complete 1 week of bedside teaching in trauma surgery during the fourth year of study. One seminar group (15–18 students) is divided in 3 smaller groups (5–6 students) for the teaching courses during the week. The obligatory course consists of 5 days with 2 teaching units of 45 min each. The course is completed with an oral examination. The topics of the courses are injuries of shoulder and knee, injuries of the spine, fractures of the ankle and foot or plastic reconstruction techniques, injuries of the upper extremity (including plastering course) and fractures of the hip and pelvis. The course evaluated in this study took place during the winter semester 2023/2024 (October 2023 to January 2024) and included 10 seminar groups with a total of 162 students.

As there were only 6 HMDs available for 15–18 students per seminar group, a total of 56 of 162 students were randomized via an anonymous allocation procedure (using https://strawpoll.com, Gregor Krambs Internet GmbH & Co. KG, Hamburg, Germany). Students had to register themselves with a pseudonymized e‑mail address and were selected by using a priority system. Each selected participant received a 1‑h technical introduction at the beginning of the course. Initially, the HMDs were handed out on Mondays and collected again on Fridays. After 2 weeks, at the request of the participants, the HMDs were handed out on Tuesdays and collected on Mondays of the following week to allow them to be used during the weekend. Device maintenance and output, management of software updates and the initial device instructions were managed with the support of the Faculty’s Skills and Simulation Center.

### Data acquisition

The participants were asked to complete a questionnaire before using the HMDs and again after the week of iVR training. The first questionnaire addressed the participants’ future career aspirations and whether they already had previous knowledge in the field of orthopedic and trauma surgery (e.g., as operational technical assistance, surgical internship). Participants were asked how many hours they were planning to use the iVR tool and why they wanted to use the HMDs. Finally, 11 items covering expectations for the iVR tool, such as technical issues, motion sickness and learning effect were included. These items were ranked on a 6-point Likert scale from 1 (total disagreement) to 6 (total agreement).

The second questionnaire evaluated how many hours the students used the iVR tool and whether their expectations were met. In addition, open-ended questions were asked concerning positive and negative aspects perceived during the use of the tool as well as technical issues that occurred.

**Fig. 1:**
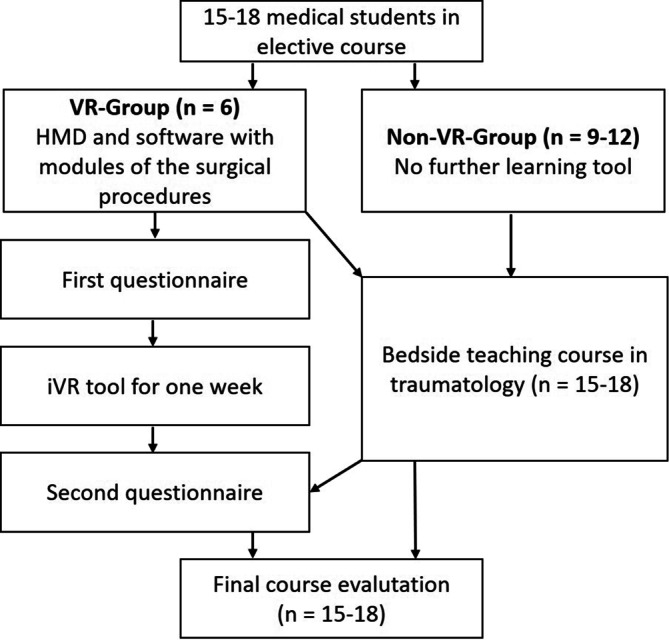
Course evaluation for each of the 10 seminar groups. *VR* virtual reality, *iVR* immersive virtual reality, *HMD* head-mounted display

At the end of the semester, all students were asked to complete the final course evaluation including a question whether the student was able to use the iVR tool. This allowed the comparison of the final evaluation of the bedside teaching between students who had the opportunity to use the iVR tool and students who had not (Fig. 1).

### Statistical analysis

Statistical analysis was performed using SPSS (IBM Corp. Released 2016. IBM SPSS Statistics for Windows, Version 24.0. Armonk, NY, USA). Data from the first and second questionnaire were compared using the Mann-Whitney U‑test or Fishers’ exact t‑test. Comparison of the students’ final evaluation was performed using the Mann-Whitney U‑test. Significance was set at *p* < 0.05.

### Data protection

No patient data were published in the lectures. All students had given informed consent to the use of anonymized data as part of the survey. To ensure anonymization, preconfigured meta-accounts were provided for the HMDs.

## Results

**Fig. 2:**
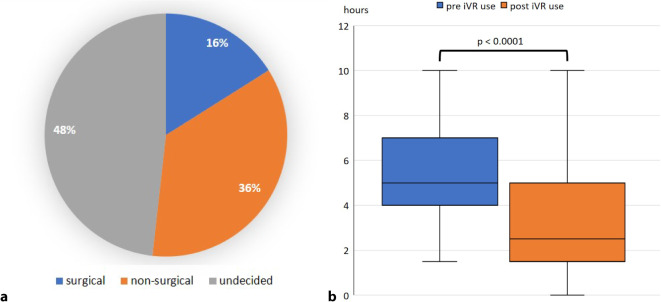
Diagram showing the career aspirations of the participants (**a**). Boxplots showing the time participants stated they wanted to invest in the iVR tool and the time they actually spent (**b**). *iVR* immersive virtual reality

The first and second questionnaire were completed by all 56 participants (mean age 24.0 years, 40 female). In total 35.7% had previous knowledge in the field of orthopedic and trauma surgery. Most of the students (36%) were undecided whether they preferred a surgical or non-surgical career (Fig. 2a). Before using the iVR tool, the participants planned to use the tool for a mean time of 5.6 h. The actual mean time of use was 3.3 h (*p* < 0.0001; Fig. 2b) during the course week.

The items covering expectations before using the iVR tool, such as technical issues, motion sickness and learning effect are shown in Table 1, whereas the re-evaluation of the same items after use of the iVR tool for 1 week or conventional bedside teaching is shown in Table 2.

**Table 1:** Expectations of the participants before using the iVR tool. The average score is the arithmetic mean ranked on a 6-point Likert scale from 1 (total disagreement) to 6 (total agreement)

Items in the first questionnaire about expectations for the iVR tool (from 1: complete disagreement to 6: complete agreement)	Average score
1) The use of VR goggles will be a useful addition to bedside teaching in orthopedic and trauma surgery	5.45
2) The use of VR goggles should be a standard in bedside teaching in orthopedic and trauma surgery	4.85
3) Virtual reality will play a role in my future career as a doctor	4.47
4) The use of VR goggles will give me a better understanding of how an operation is performed	5.24
5) I have the confidence to assist in a real surgery	4.13
6) I am interested in surgical subjects	4.76
7) I expect that the 1‑h introduction to the technology of VR goggles is sufficient	5.22
8) I would like to use the VR goggles exclusively under the guidance of a tutor as part of a seminar use	2.24
9) I do not expect any problems with the technical equipment	4.28
10) I expect to be overwhelmed with the various simulation surgeries of the VR goggles	3.00
11) I expect side effects such as motion sickness when using VR goggles	3.26

**Table 2:** Evaluation of the participants after using the iVR tool. The average score is the arithmetic mean ranked on a 6-point Likert scale from 1 (total disagreement) to 6 (total agreement).

Items in the second questionnaire about experiences with the iVR tool (from 1: complete disagreement to 6: total agreement)	Average score
1) The use of VR goggles was a useful addition to bedside teaching in orthopedic and trauma surgery	5.45
2) The use of VR goggles should be a standard in bedside teaching in orthopedic and trauma surgery	5.07
3) Virtual reality will play a role in my future career as a doctor	4.45
4) The use of VR goggles gave me a better understanding of how an operation is performed	4.87
5) I have the confidence to assist in a real surgery after using the VR goggles	3.67
6) Trying out the VR goggles has increased my interest in surgical subjects	4.45
7) The introduction to the technology of VR goggles was sufficient	5.38
8) I would like to have used the VR goggles exclusively under the guidance of a tutor as part of a seminar use	2.33
9) There were no technical issues with the equipment	4.20
10) I was overwhelmed with the various simulation surgeries of the VR goggles	2.64
11) I developed side effects such as motion sickness when using VR goggles	3.18

**Fig. 3:**
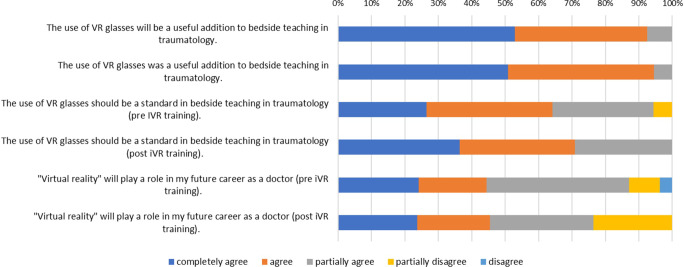
Bar graph showing the participants expectations before using the iVR tool and their evaluation after using the iVR tool regarding the role of virtual reality in bedside teaching and their future career as doctors. *iVR* immersive virtual reality

Most of the participants expected and later agreed that iVR tools can be a valuable add-on to conventional bedside teaching in orthopedic and trauma surgery. In addition, the great majority expect virtual reality to play a role in their future career as physicians (Fig. 3).

All students expected that the use of iVR will give them a better understanding of how a surgical procedure is performed. After using the iVR tool 7.3% of participants agreed on this matter only partly. The confidence to assist in a real surgery decreased to some degree from 68.5% to 58.2%. The confidence to assist in real surgery was higher in participants with previous knowledge in surgery (*p* = 0.0004), whereas there was no statistically significant difference in confidence between male and female students (*p* = 0.155).

**Fig. 4:**
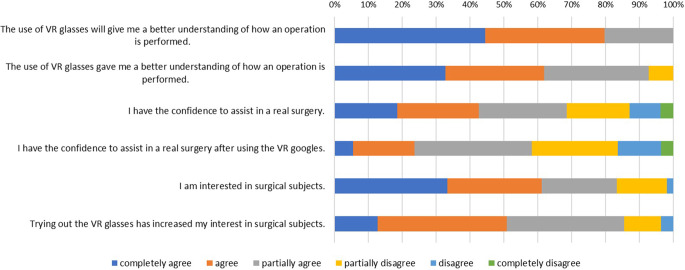
Bar graph showing the participants’ expectations before using the iVR tool and their evaluation after using the iVR tool regarding the role of virtual reality in understanding surgical performances, confidence to assist in a real surgery and their general interested in surgical subjects. *iVR* immersive virtual reality

The participants’ interest in surgical subjects was slightly increased from 83.3% to 85.5% after using the VR goggles in the course (Fig. 4). There was no statistically significant difference between male and female students and their interest in surgical subjects (*p* = 0.253). Students with previous knowledge in surgery stated to have less interest in surgical subjects than students without previous knowledge (*p* = 0.04).

**Fig. 5:**
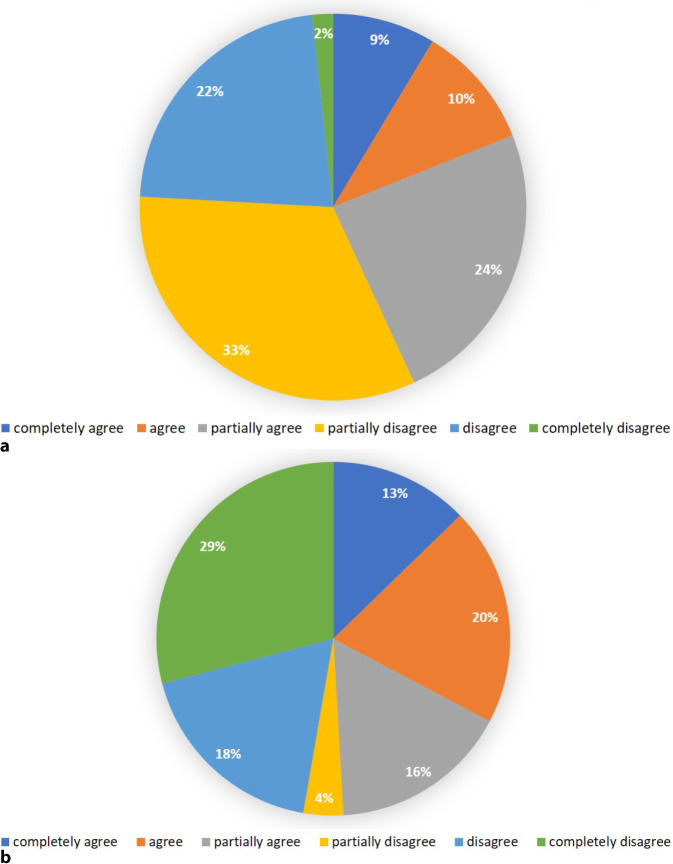
Diagrams showing the expectation to developed motion sickness before the iVR training (**a**), and the development of symptoms of motion sickness after using the VR goggles (**b**). *VR* virtual reality

**Fig. 6:**
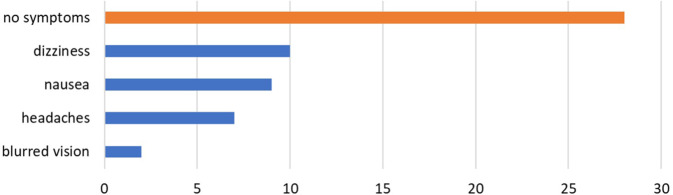
Diagram of the symptoms that were described by the participants after using the VR goggles. *VR* virtual reality

**Fig. 7:**
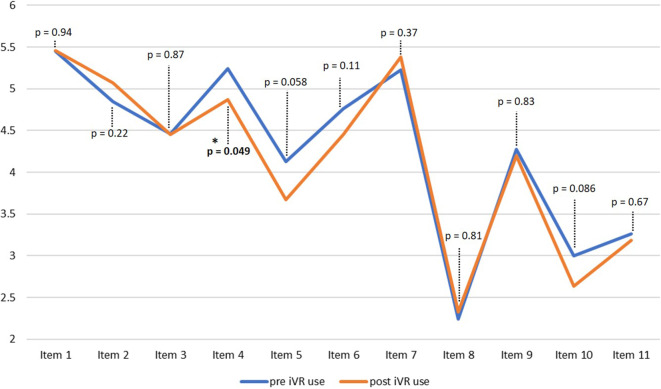
Line diagram comparing the participants’ expectations before using the iVR tool and their evaluation after using the iVR tool for all items asked in the first and second questionnaire. *iVR* immersive virtual reality

Regarding motion sickness, 43% of the participants expected to develop symptoms to some degree (Fig. 5a). After the use of the VR goggles 12% of participants strongly agreed and 20% agreed to have developed symptoms of motion sickness, whereas 29% of students strongly and 22% (partly) disagreed to have developed any symptoms (Fig. 5b). The most frequent specified symptoms were dizziness (37.0%), nausea (33.3%) and headaches (25.9%). Some students developed more than one of the symptoms (Fig. 6). There was no significant difference between males and females for the development of symptoms (*p* = 1.000). Overall, the expectations were met for almost all items asked, showing no significant differences between participants’ expectations (questionnaire 1) and their experiences after using the iVR tool (Fig. 7). The only exception not met was the expectation that use of the VR goggles will give the participants a better understanding of how an operation is performed. For this item, the expectation decreased from a mean score of 5.2 to 4.9 after iVR training (*p* = 0.049).

In the open-end responses, the participants positively emphasized the advantages of learning practical skills interactively compared to the otherwise mainly theoretical curriculum. Especially the opportunity to learn and train skills at an own pace was appreciated. The participants also mentioned that iVR was a good tool for improving visuospatial skills due to the realistic in situ representation of anatomical structures.

The increased motivation to acquire new knowledge by using iVR as an exciting and fun learning tool was particularly highlighted. Regarding the PrecisionOS software, the students emphasized the lifelike reproduction of an operating scenario and the versatility of application. Negative aspects mentioned by the participants were technical difficulties, language barriers (software in English) and a lack of setting options for left-handers. Some participants stated that sometimes even after several restarts the software did not work and therefore the training had to be stopped. A total of 8 participants (14.3%) stated that they lacked the knowledge to comprehend the procedure of some operations. Except for one participant, none of these students had previous experience in the field of orthopedic and trauma surgery. It was frequently stated (*n* = 8) that the usage interval of 1 week was not sufficient and the participants would have liked more time with the iVR headsets. The reasons given for this were other commitments such as preparing for examinations and other courses.

One repeatedly mentioned aspect (*n* = 7) was insufficient haptic feedback. Participants emphasized that more precise haptic feedback would have improved the users experience of the iVR tool, especially in arthroscopic procedures.

At the final questionnaire of the students at the end of the academical term, only 33 students participated, of whom 19 (57.6%) had received an HMD. Of the students who received an HMD 88.9% stated that it was a useful addition for bedside teaching. Students who received an HMD rated the bedside course (from 1 best to 6 worst) better (1.67 ± 0.74) than students who did not receive an HMD (2.14 ± 0.8) without statistical significance (*p* = 0.184).

## Discussion

The primary objective of this research was to elucidate how an immersive virtual reality (iVR) tool could be integrated into medical student curricula, specifically in orthopedic and trauma surgery. Whilst other disciplines have a plethora of VR tools in medical education for non-residents [5], in orthopedic surgery, only one study reported the usage of VR training for medical students, introducing students to an intensive care unit and prehospital care [13]. This is in line with the observation that the use of VR simulation in orthopedic training programs is less common than in other surgical specialties [1, 36]. The need for alternatives for cadaver training was especially highlighted by the COVID-19 pandemic [30]. Recent studies could already show that VR can assist in the teaching of anatomy in undergraduate students [23, 33]. Currently most of the systematic reviews focus on VR teaching for physicians and residents. The impact of VR training on medical students is underrepresented in the current literature [16]. While most evidence for iVR training efficacy exists for residents, Mao et al. found no obvious variations in the efficacy of iVR for surgical skills training between medical students and residents [21]. This could be explained by the limited access to hardware and software. The approximate cost of iVR tools vary between US $1.500 and US $8.000, depending on the institutional licensing agreements [6, 14, 19]. Furthermore, factors such as device maintenance and output, management of software and updates as well as device instructions must be considered [21]. For these reasons it was not possible to provide access to the iVR training to all 162 students who were enrolled in the obligatory course in the present investigation. Device maintenance, software management and device instructions were managed with support of an assistant employed especially for this purpose and the Faculty’s Skills and Simulation Center. In the present study it could be shown that iVR tools can be effectively implemented in curricular teaching in orthopedic and trauma surgery, although access had to be limited in the present set-up. Most participants stated that a 1h instruction to the technology of the VR goggles was sufficient. Thus, there may be some cost-effectiveness in iVR training as it allows the training of skills by students in the absence of teaching personnel after a structured introduction. This is in line with the findings of Lohre et al., who estimated iVR training to be up to 34.1 times more cost-effective than traditional training methods [20].

Participants feedback was predominantly positive: after iVR training, all participants agreed that VR goggles are a useful addition and should be a standard in bedside teaching in orthopedic and trauma surgery. This is in line with current literature that shows predominantly positive user feedback of surgical residents evaluating the usability of different iVR simulators [4, 14, 20]. In a training model for neurosurgical procedures utilizing iVR by Atli et al., all participating medical students evaluated the course as valuable learning experience and iVR as useful learning tool [2]. The results of the present study and the evaluation by Atli et al. indicate favorable advantages and high acceptance of iVR in medical education especially in surgical disciplines [2]. In the present setting, the participants especially praised the possibility of flexible learning times. This is in line with other reports as independence from time constraints is often described as positive aspect of online teaching [27, 28]. Another aspect appreciated by the students was the opportunity to view anatomical structures for visualization during iVR training, which led to a subjective improvement in visuospatial abilities. Virtual reality has been shown to be effective in improving students’ understanding and retention of knowledge of surgical anatomy [23, 24, 33] and improving visuospatial skills [3, 19].

Although almost all expectations of the participants were met, many students reported that the operations were difficult to manage without haptic feedback. For this reason, especially arthroscopic procedures might have appeared more difficult than in reality. Some of the participants mentioned a lack of comprehension due to insufficient knowledge in relation to procedural steps in surgical procedures. This might be explained by the present set-up: as shown by Hew et al., it can be useful to give instructions on complex topics in advance to maximize learning success at a later point [12]. Therefore, annotated videos and supporting educational material could be useful. A frequently observed downside of iVR training is the occurrence of motion sickness [4, 13, 15], which is caused by a mismatch between the sensory systems by using HMDs [13]. Barré et al. reported slight to moderate symptoms of motion sickness in 30.0% of their participants [4], whereas it was 23% in the study by Holla et al. [13]. In the present study, 49.0% showed symptoms of motion sickness, mainly dizziness, nausea, and headaches. Although it is suggested that females are more likely to develop motion sickness than males [11, 31], this could not be observed in the present study. This may be explained by the HMD used, which allowed different levels of interpupillary distance (IPD) to be set. Stanney et al. were able to show that an inadequate IPD is a primary driver of gender differences in cybersickness [31]. Nevertheless, the occurrence of motion sickness remains a limitation of iVR in medical education, although postural discomfort has been shown to decrease with increased practice [4].

This study has certain limitations: (1) due to structural limitations, only one third of the students enrolled in the curricular bedside teaching course in traumatology could be equipped with the HMDs. (2) The outcomes are solely descriptive. Therefore, a comparison regarding the influence of iVR training on the medical student’s performance in the course could not be documented. (3) Only 33 students participated in the final course evaluation, so only limited data could be obtained. (4) Influences on the occurrence of motion sickness (e.g. experiences in gaming or habituation effects) were not surveyed.

## Conclusion

Immersive virtual reality can be easily implemented into the undergraduate medical curricula. The high expectations of the participants were fulfilled and students would favor iVR to be a regular part of the obligatory courses in orthopedic and trauma surgery. Participants spent a mean time of 3.3 h using the iVR tool in addition to regular course content. Motion sickness occurred in half of the cohort.

## Data Availability

The datasets used and/or analyzed during this study are available from the corresponding author upon reasonable request.

## Abbreviations

3D: 3‑dimensional
ACL: Anterior cruciate ligament
COVID-19: Coronavirus disease 2019
HMD: Head-mounted display
IPD: Interpupillary distance
iVR: Immersive virtual reality
VR: Virtual reality

## Declarations

Due to the survey-based study design, no approval of the ethics committee of the University Hospital of Leipzig was needed.
